# The potential for the use of leghemoglobin and plant ferritin as sources of iron

**DOI:** 10.1515/biol-2022-0805

**Published:** 2023-12-13

**Authors:** Michał Świątek, Adrianna Antosik, Dominika Kochanowska, Paweł Jeżowski, Krzysztof Smarzyński, Aneta Tomczak, Przemysław Łukasz Kowalczewski

**Affiliations:** Ekosystem-Nature’s Heritage Association, Institute of Microbial Technologies, Al. NSZZ Solidarność 9, 62-700 Turek, Poland; Institute of Chemistry and Technical Electrochemistry, Poznan University of Technology, 4 Berdychowo St., 60-965 Poznań, Poland; InnPlantFood Research Group, Poznań University of Life Sciences, 31 Wojska Polskiego St., 60-624 Poznań, Poland; Department of Biochemistry and Food Analysis, Poznań University of Life Sciences, 48 Mazowiecka St., 60-623 Poznań, Poland; Department of Food Technology of Plant Origin, Poznań University of Life Sciences, 31 Wojska Polskiego St., 60-624 Poznań, Poland

**Keywords:** plant-based diets, sources of iron, LegH, iron deficiency

## Abstract

Iron is an essential component for the body, but it is also a major cause for the development of many diseases such as cancer, cardiovascular diseases, and autoimmune diseases. It has been suggested that a diet rich in meat products, especially red meat and highly processed products, constitute a nutritional model that increases the risk of developing. In this context, it is indicated that people on an elimination diet (vegetarians and vegans) may be at risk of deficiencies in iron, because this micronutrient is found mainly in foods of animal origin and has lower bioavailability in plant foods. This article reviews the knowledge on the use of leghemoglobin and plant ferritin as sources of iron and discusses their potential for use in vegetarian and vegan diets.

## 1 Introduction

The type of diet is one of the main factors affecting human health. It is indicated that a diet rich in meat products, especially red meat and highly processed products, processed products, increases the risk of developing lifestyle diseases such as circulatory system diseases, heart diseases, and cancer. A diet rich in plant products is considered an example of a health-promoting diet. It is estimated that switching to plant-based diets globally could reduce the risk of premature death from non-communicable diseases by 18–21% and additionally reduce greenhouse gas emissions by 54–87% [1].

In contrast, vegetarians and vegans may be at risk of deficiencies in vitamin B_12_, vitamin D, iron, zinc, and calcium deficits because these micronutrients are found mainly of animal sources or have lower bioavailability in plant foods. Although plant-based diets are described as healthier for the body’s health, they should be balanced to provide the appropriate amount of nutrients needed for a healthy life every day [2,3,4,5,6].

One of the most common deficiencies occurring among human on plant-based diet is iron deficiency (ID) in serum. The reason for such a problem originated mainly in the lower bioavailability of iron from plant sources and the presence of inhibitors of iron absorption in plant-based food. Some plant-based diets are significantly restricted for the application of ingredients or technological processes, including materials from animals in food manufacturing. All those factors, as well as progress in plant-based food technologies, contribute to the development of knowledge about the possibilities of enriching the human diet with alternative iron sources in populations at risk of ID due to the exclusion of animal-based food from the diet.

The purpose of this review is to present recent knowledge about ID and its connection with plant-based diets and information about alternative iron sources such as leghemoglobin (LegH) and (plant)ferritin as a substance that are considered promising in plant-based food fortification.

## 2 Iron in the diet and its impact on human health

Iron is classified as a microelement that is necessary for the proper functioning of human cells. In the human body, it is a basic component involved in the transport and binding of oxygen and a cofactor of many enzymes, but it also plays an important role in the body’s immune processes. About 2/3 of the human body’s iron is found in hemoglobin, 1/4 is in the liver in the form of ferritin (FT) and hemosiderin, with the remaining sources scattered in the cytoplasm of muscle cells in the form of myoglobin [7,8].

ID is a burden contributing to the development of diseases in a significant part of the population, and the most common disease associated with ID is anemia (iron deficiency anemia, IDA). According to statistical data, the burden of IDA concerns up to 1.2 billion of the population [8]. Women (in the premenopausal period and pregnant women), but also infants and children up to 5 years of age and teenagers, are particularly susceptible to the occurrence and consequences of anemia [8]. The occurrence of ID and IDA also depends on social and demographic factors such as race/ethnicity, socioeconomic status, religion, eating habits, and diet [9,10]. Due to the high risk of IDA, preventing anemia is one of the tasks of the WHO, which aims to reduce its incidence among women by 50% [11].

ID may cause symptoms in the presence or absence of anemia or may be asymptomatic. Typical symptoms include fatigue, drowsiness, decreased concentration, dizziness, tinnitus, and pallor. In susceptible individuals, ID predisposes to restless legs syndrome [12]. Other symptoms include alopecia, dry hair or skin, and glossitis [13]. ID and anemia can also exacerbate symptoms and worsen prognosis in conditions including heart failure and coronary artery disease [14]. Preoperative anemia increases the risk of blood transfusion and is correlated with a negative course of recovery and postoperative mortality [15]. Even asymptomatic anemia has negative consequences, including impaired physical fitness, neurocognitive development of the child, and the course of pregnancy [16].

However, it is not only ID that is harmful to our health. The most frequently cited causes of iron overload (IO) are red blood cell transfusion, increased gastrointestinal iron absorption, repeated whole blood transfusions, excessive iron supplementation, chronic hepatitis, or hereditary hemochromatosis [17]. The toxicity of excessive iron content leads to serious side effects such as cardiac dysfunction, including arrhythmia and cardiomyopathy [18], liver cirrhosis, liver cancer, hepatitis [19], delayed puberty, impotence, infertility [20], metabolic disorders [21], and development of neurodegenerative diseases, e.g. Alzheimer’s [22].

The primary source of iron is the diet. For a healthy body, without metabolic disorders, achieving an appropriately balanced diet is not difficult in developed countries; therefore, ID in such populations, apart from particularly susceptible groups (children under 5 years of age, pregnant women), is usually the result of metabolic disorders, blood loss (e.g. due to heavy menstruation), as well as the ingestion of a diet with a limited supply of nutrients (e.g. plant-based diets) [23].

Iron in the human diet occurs in two forms: heme iron in organic compounds and non-heme iron occurring in inorganic compounds such as Fe^2+^ and Fe^3+^ ions. The different forms of iron found in food differ in their bioavailability. The absorption of heme iron reaches 15–35% and is only slightly dependent on the presence of inhibitors in the consumed food [24]. Heme iron has the highest bioavailability, and its best sources are meat, fish, and seafood. In a diet including products of mixed origin (plant and animal), heme iron usually constitutes approximately 10–15% of the daily iron intake [25]. The best sources of non-heme iron are seeds, grains, nuts, and dark green parts of leafy vegetables [25]. Non-heme iron occurs in various chemical forms (Fe^2+^ and Fe^3+^), which significantly affects its absorption, typically reaching levels of 2–20%, with the ferrous ion (Fe^2+^) being the form with the highest absorption [26]. The forms of iron present in food are low-molecular compounds, such as citrate, phosphate, phytate, oxalate, and iron hydroxide, and high-molecular compounds, such as FT (a protein that combines thousands of iron ions in the mineral core). A specific organic compound that is a source of iron in the diet of infants is lactoferrin (iron transport protein), present in breast milk and infant formulas [27,28].

Iron absorption is strongly dependent on the level of iron in the body. In case of its deficiency, iron absorption usually increases 10-fold, and this applies to both heme and non-heme iron. Moreover, the absorption of non-heme iron is influenced by both its inhibitors (phytic acid, polyphenols, calcium, milk and egg proteins, and albumins) and enhancers such as ascorbic acid and muscle tissue proteins [29]. The absorption of iron is enhanced by MFP (meat, fish, poultry) protein factors, which are present in food of animal origin such as meat, fish, and poultry products. The role of MFP factors in improving the absorption of iron supplied with food of plant origin has been demonstrated. The use of poultry, beef, or fish in the diet improves the absorption of non-heme iron two to three times compared to the absorption from a meal containing the protein equivalent of egg white albumin [30,31,32].

## 3 Iron in plant-based diets

Nowadays, diets that limit or completely exclude the consumption of meat products are becoming more and more popular. They are most often used by young people, especially women. Their motivation to limit the consumption of meat products is health (reducing the risk of lifestyle diseases), ethics, and the environment. A diet takes different forms depending on the type of products that are not consumed (Table 1). The lightest form is flexitarianizm, which involves limiting meat consumption to a minimum [33].

**Table 1:** Types of plant–based diets; the food categories included in each type of diet have been stated in table

	Fruits	Vegetables/grains/legumes	Dairy	Eggs	Meat/poultry	Fish
Vegetarian	P	P	P	P	A	A
Lacto-ovo vegetarian	P	P	P	P	A	A
Lacto vegetarian	P	P	P	A	A	A
Ovo vegetarian	P	P	A	P	A	A
Vegan	P	P	A	A	A	A
Flexitarian	P	P	P	P	P	P
Pesco-vegetarian	P	P	P	P	A	P

P – present, A – absent.

There is a variety of diets that eliminate animal products to different extents. Vegetarians are people who do not eat meat, including poultry, fish, seafood, and products containing ingredients derived from the processing of these raw materials. Lacto-ovo vegetarians exclude the consumption of meat, but their diet includes milk, eggs, and products derived from their processing. Lacto-vegetarians also exclude the consumption of eggs, unlike vegetarians, whose diet does not include milk and dairy products. Among all these variants of the vegetarian diet, intermediate variants are described resulting from different scopes of exclusion of animal products (e.g. semi-vegetarianism/flexitarian, pollotarian, pescatarian). The vegan diet does not include any products obtained from raw materials of animal origin [34,35,36]. The use of vegetarian and vegan diets (due to the richness of phytocomponents with health-promoting effects) is believed to provide attractive health-promoting properties, such as the prevention of cancer or ischemic heart disease, a beneficial effect on the reduction of BMI, low levels of total cholesterol, LDL cholesterol, and glucose [37,38].

According to studies comparing the iron content in the diet of vegetarians, lacto-vegetarians, and non-vegetarians (both among the Seventh-day Adventists population) and the diet of people consuming meat products, the type of diet and the extent to which meat and meat products are eliminated from the diet have a minimal impact on the supply of iron in the diet of the Western population. The average three-day supply of iron in the compared diet variants was 18.0 ± 1.6, 14.2 ± 0.8, 14.4 ± 0.9, and 16.1 ± 1.1 mg Fe/day, respectively [39]. The intake has been computed by the nutrient data bank providing Fe content. In contrast, assessing iron supply solely on the basis of the total Fe content in the diet is not an accurate way to assess the potential of meals to meet nutritional needs for this element. This is due to the different forms and bioavailability of iron depending on the type of food. Therefore, although a vegetarian diet provides similar amounts of iron to a diet including animal products, there are significant differences in the availability of iron for absorption, resulting from the different forms of iron (heme and non-heme) and the amount and profile of the supplied inhibitors and absorption enhancers [40,41]. The main sources of phytic acid, which is an inhibitor of iron absorption, are whole-grain products, legumes, lentils, and nuts [42,43]. Polyphenols, also limiting iron absorption, are additionally supplied with coffee, tea, red wine, and spices [25,44]. Although these factors may suggest a lower degree of meeting iron needs among people on a diet eliminating animal products, the data on this subject are not clear, and future research should include not only intake of Fe but serum analysis also.

The parameter considered appropriate to assess the degree to which the body’s iron needs are met is the analysis of serum iron. Other indicators include hemoglobin, hematocrit, and iron-binding capacity. Regardless of the selected indicators, there are no clear results on the impact of diet on the degree to which the body’s iron needs are met. The described cross-sectional studies often compare iron/FT content in people on an elimination diet and people who include meat in their diet. Some of these comparisons do not indicate statistically significant differences [41,45,46,47]. Others report significantly lower iron stores in people on a vegetarian diet [48,49,50,51]. There are also reports of a higher incidence of ID and IDA among these people, especially in women [48,52,53].

## 4 New sources of iron for use in plant-based diets

One of the options for supplementing iron in people on a diet eliminating animal products is the use of supplementation. However, no direct data indicates the achievement of long-term beneficial effects from such an action. In this context, there is information suggesting that iron supplementation limits the efficiency of iron absorption from meals, requires long-term use to improve the FT content in serum (observations conducted among women with low iron resources), and may additionally leads to an increase in oxidative stress due to the presence of unabsorbed iron ions in the intestine [54,55,56]. Therefore, it is suggested that a good solution is to enrich food with easily digestible iron and thus meet the demand for this element. Among the sources of iron, food producers most often use iron salts, which, however, can cause negative effects on the organism, e.g. by causing and exacerbating stomach inflammation [54,55]. Therefore, new sources of plant iron are being sought, which will be devoid of negative implications, but with high bioavailability. LegH and FT are described as the most promising.

### 4.1 Leghemoglobin

LegH is an oxygen-binding phytoglobin with a molecular weight of 16 kDa, first identified in the root nodules of legumes [56]. This symbiotic hemoglobin is composed of protoporphyrin IX (a heme group) and globin (a single peptide). The amino acid sequence of globin depends on the species of legume, while the heme group remains unchanged, regardless of the plant species and bacterial strain. The complex mixture of heme proteins contains two monomeric main components (LegH-a and c) and two less well-characterized minor components (LegH-b and d). Studies using ion exchange chromatography have shown that both LegH-c and LegH-d are mixtures of at least two heme proteins. The ratio of the amounts of various components of LegH depends on the age of the nodules. In the case of young root nodules, a characteristic higher content of LegH-c compared to LegH-a was observed. LegH components can also be classified, using electrophoresis, into fast-moving components (LegH-F), such as LegH-c1 and LegH-c2, and slower-moving components (LegH-S), such as LegH-a [57,58].

This hemoprotein typically consists of seven or eight helical segments A–H, with protoheme (an iron porphyrin molecule) inserted between helices E and F. Between the heme and the E-helix, there is a space called the distal pocket, which allows oxygen to approach and coordinate in a manner reversible with heme iron [59]. Helix D is present only in the β chain of hemoglobin and myoglobin; it is not present in the distal heme pocket. This is replaced by a longer interhelical CD region connecting to the critical E helix, ensuring helix mobility. Research indicates the same structure of the animal and plant hemoglobin gene, which during evolution underwent genetic rearrangement through the loss of an intron and led to changes in the amino acid sequence in approximately 80% of positions [57].

**Figure 1:**
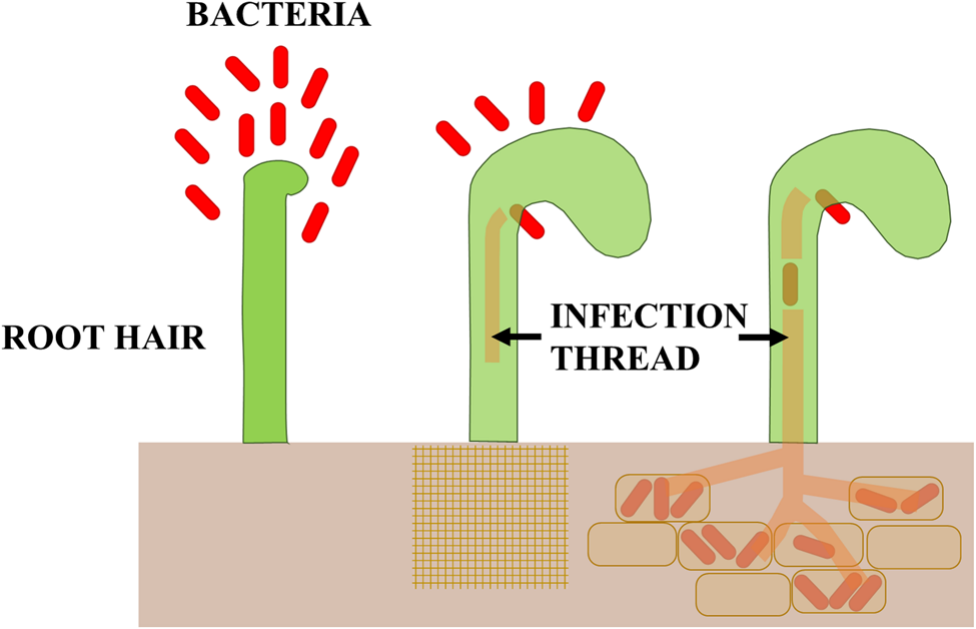
Scheme of overgrowing the roots of legume plants by bacteria of the *Rhizobium* genus.

The production of LegH is possible through symbiosis between the bacteroid and the plant (Figure 1). Its synthesis begins immediately after nodulation initiation and just before nitrogenase synthesis. Symbiosis occurs in root nodules resulting from bacterial infections, and a meristem is formed in the root cortex. It divides to give rise to two types of cells – cells filled with bacteroids and smaller interstitial cells devoid of bacteroids. As a result of this division, in mature legume nodules, a structural division into three separate zones can be observed – the outer cortex, the central zone, and the inner cortex [57,60]. Endodermis – the cellular layer separating the outer and inner cortex – acts as a physical barrier to oxygen diffusion. There are no free bacteroids in the cell cytoplasm; after infection and throughout the entire period of symbiosis, bacteroids are surrounded by a symbiosomal membrane (peri-bacteroid) [61].

**Figure 2:**
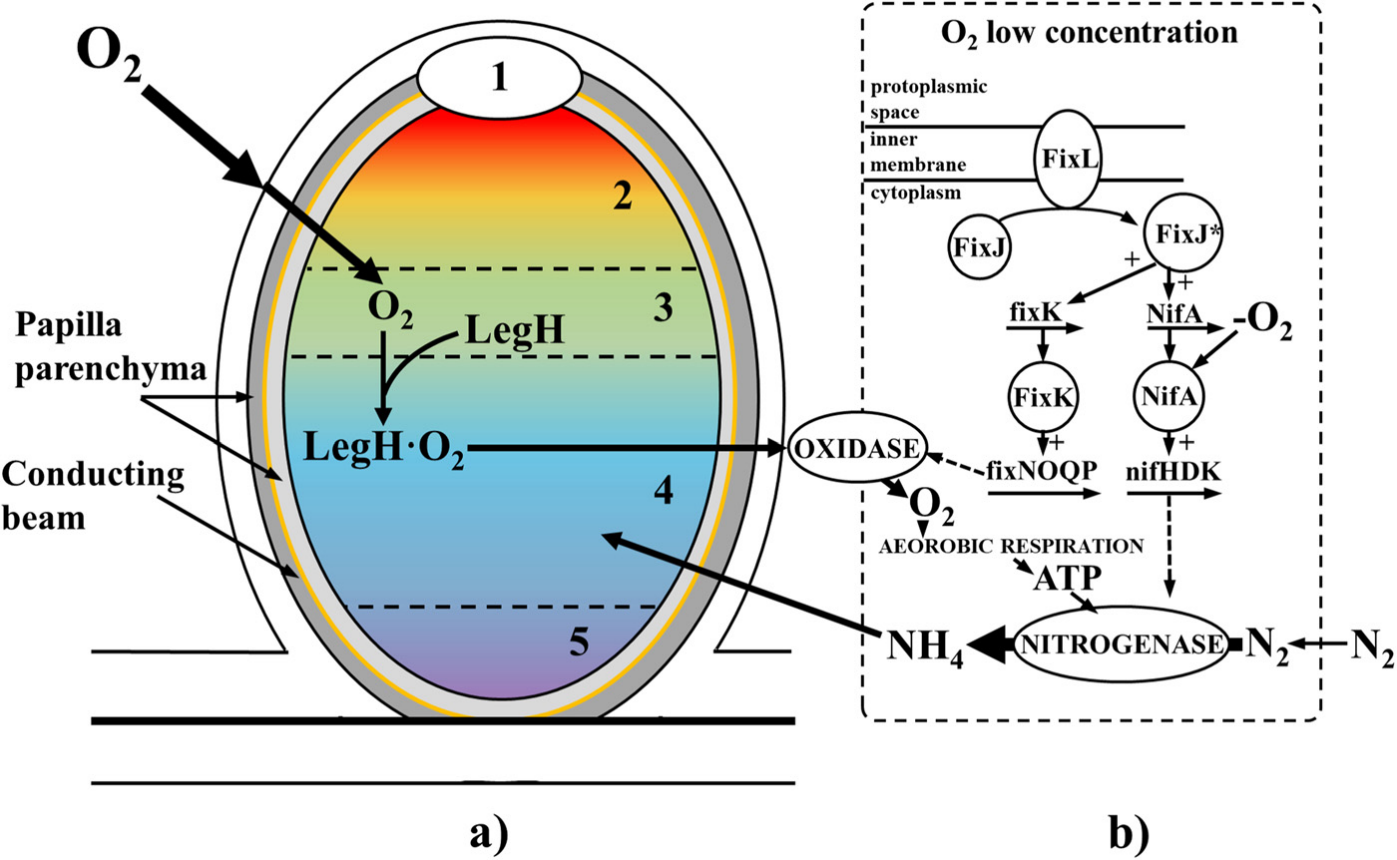
Participation of LegH in the regulation of bacterial gene expression and nitrogenase functioning (prepared based on [65], available under the CC-BY license). (a) Diagram of the root nodule structure with distinction of the zones formed during the nodule development: 1 – nodule meristem, 2 – infection zone, 3 – intermediate zone, 4 – nitrogen fixation zone, 5 – aging zone. (b) Simplified model of the regulation of some bacterial genes as a result of lowering the oxygen concentration in the infection zone. *LegH* genes are expressed in the infection, intermediate, and nitrogen fixation zones. LegH transfers oxygen to bacterial oxidases, and these, in turn, to the respiratory chain, enabling the production of ATP in conditions of low oxygen concentration.

Characteristic properties of LegH include the ability to bind oxygen, similar to human hemoglobin. It binds oxygen in the nodule and establishes sufficient oxygen levels for rhizobia to respire and create ATP. It is responsible for providing bacteria with a sufficient amount of oxygen and protecting nitrogenase – an enzyme that catalyzes the environmental conversion of nitrogen to ammonia – from denaturation. Nitrogenase is an enzyme that is particularly sensitive to oxygen and is irreversibly deactivated when exposed to atmospheric oxygen. Oxygen diffusion through the dense nodule tissue would not be sufficient to meet ATP demand in the absence of LegH. For LegH to release oxygen into the bacteroid respiratory chain, its oxygen affinity must be approximately ten times lower than the oxygen affinity of bacteroid oxidase. This allows oxygen to flow through the tissue and limits the possibility of free oxygen accumulating on the surface of the bacteroid. LegH gives the nodules, which effectively fix nitrogen, their pink color. The presence of LegH in the root nodules of legumes is therefore necessary for the proper functioning of nitrogenase (Figure 2). It takes up as much as 40% of the total share of soluble proteins in papillae [57,62,63]. In addition, LegH may also influence plant metabolism, and some studies suggest that it may influence plant defense responses against pathogens. LegH is completely absent in the intercellular spaces or above other cellular organelles. The concentration of LegH in the root nodules of legumes ranges from 1–5 × 10^4 ^M [57,64].

The process of isolating LegH varies depending on the host plant species. The two most commonly used legumes for the production of LegH are soybeans and lupine. The first isolation methods were described already in 1980 [66]. The possibilities of using LegH in the diet are mainly related to its ability to bind oxygen. LegH has great potential as an extremely attractive component of vegetarian and vegan diets. It can be successfully used as an ingredient imitating the sensory properties of meat (texture, taste, smell) despite its plant origin, providing essential nutrients (mainly amino acids, saturated fats, sodium, and iron). The bioavailability of LegH preparations has been studied in animal models and *in vitro* Caco-2 cell models with different results. However, the Caco-2 model seems to be more reliable in studying the rate of bioavailability in humans. The study of Proulx and Reddy describing the effects of food supplementation with LegH indicates that the bioavailability of hemoglobin from soy root nodules is similar to that of heme iron from animal sources. When tested in the Caco-2 model, the relative biological values exhibited for LegH 27 ± 6% and bovine hemoglobin 33 ± 10% higher bioavailability than FeSO_4_ (*P* < 0.05) and with no difference between them [67]. However, future work is needed to confirm the potential ability of soy LegH in plant-based meat alternatives to contribute to iron status *in vivo* in humans [68]. Introducing legumes containing LegH into the diet can contribute to sustainable development, because growing plants is ecological and has less impact on the environment compared to animal breeding [69,70].

However, the production of LegH for industrial purposes is a process based on genetically modified organisms. The genes responsible for the production of LegH are introduced into plants that do not naturally contain this protein. In July 2016, a Californian company developing substitutes for meat products – Impossible Foods – introduced its flagship product – a vegan alternative to a beef hamburger [71]. In addition to improving taste and providing aroma, soy LegH protein is an excellent source of iron, similar to myoglobin, which is a source of iron in meat [72]. Currently, heme iron in the human diet is almost exclusively iron of animal origin. For the production of meatless burgers, heme is produced by a safe recombinant method using *Pichia pastoris* yeast genetically modified with the soy LegH gene and using a fermentation process. The yeast releases the globin; the next step is filtration until a minimum of 80% purity of LegH is obtained. It is a routine recombinant method used to produce enzymes or even drugs [73,74].

A number of studies were carried out to assess the safety of LegH obtained using *in silico*, *in vivo*, and *in vitro* methods. Some of them are presented in Table 2. Studies have confirmed that LegH produced in yeast has little or no allergenic potential and does not show mutagenicity in the bacterial reverse mutation test (Ames test) or clastogenicity in the chromosome aberration test under tested *in vitro* conditions [75]. To exclude potential systemic toxicity, a 28-day *in vivo* study was also performed introducing prepared LegH into the rat diet, and no negative impact on the health of females or the estrus cycle was observed. The results of all tests performed suggest that the produced protein does not raise toxicological concerns under the tested conditions. This preparation is safe and suitable for human consumption as an ingredient of a simulated meat product, with a maximum soy protein content of 0.8% [75]. The company producing the mentioned burger asked the US Food and Drug Administration (FDA) for permission to use recombinant soy LegH as an analogue of animal hemoglobin in its products [71]. The FDA granted approval in 2021, and although challenged, the consent was upheld by a decision of the federal appellate court in San Francisco [76,77]. This product has been recognized as a safe food additive (GRAS). Research has confirmed that this vegan alternative to ground beef is a better source of protein, is lower in total fat than a similarly sized beef cutlet, and contains no cholesterol. The developed preparation is produced using *Pichia pastoris* bacteria, whose genome has a gene encoding a given protein incorporated into it, which is why it is classified as genetically modified food in food law. Therefore, the introduction of food with the addition of LegH is not simple in legal terms, especially in European Union countries. Many countries restrict the introduction of genetically modified foods, and European Union regulations are considered the most restrictive [78]. Therefore, the introduction of LegH into wider production seems highly doubtful, which is why research is ongoing on other sources of iron.

**Table 2:** Review of selected studies on the allergological and/or toxicological safety of LegH

Aim of research	Methods	Conclusions	Refs.
This article focuses on the safety, by means of allergenic potential of *Pichia*-derived 83 proteins, from a new simplified manufacturing process to produce LegH Prep	– Shotgun proteomics – Mass spectrometry (IPMS) – Top-down proteomics analysis – Characterization of feme cofactor – Bioinformatics search – *In vitro* pepsin digestibility study of LegH	A weight-of-evidence approach was used to determine the allergenicity of LegH Prep. No significant matches were found except for the highly evolutionarily conserved GAPDH protein. Results showed that there is an unlikely risk of cross-reactivity between LegH Prep and GAPDH	[72]
To check whether food containing recombinant soy LegH produced by *Pichia* sp. is allergenic or toxic	– Sequence databases and bioinformatics search strategies (sequence of LegH and the 17 *Pichia* host proteins; AllergenOnline (AOL) version 2016 (v16); BLASTP in NCBI Entrez protein database; *In vitro* pepsin digestibility study) – Pepsin activity test – Determination of system’s limit of detection (LOD) – - Pepsin digestion resistance tests of LegH and three control proteins	The results demonstrate that foods containing recombinant soy LegH produced in *Pichia* sp. are unlikely to present an unacceptable risk of allergenicity or toxicity to consumers	[79]
Safety evaluation of soy LegH protein preparation derived from *Pichia pastoris*, intended for use as a flavor catalyst in plant-based meat	– Bacterial reverse mutation assay (Ames test) – Chromosome aberration assay in human peripheral blood lymphocytes – 14-day dietary palatability and range finding study in rats – 28-day dietary feeding study in rats – 28-day investigative study with a 14-day predosing estrous cycle determination	LegH Prep was nonmutagenic and nonclastogenic in each test. There were no mortalities associated with the administration of LegH Prep in a 28-day dietary study in male and female Sprague Dawley rats. Collectively, the results of the studies presented raise no issues of toxicological concern with regard to LegH Prep under the conditions tested	[75]
Design of *P. pastoris* for highly secretory production of LegH without exogenous addition of expensive precursors	CRISPR/Cas9 mediated genome editing methods	Increased the secretion of LegH by more than 83-fold, whose maximal LegH titer and heme binding ratio reached as high as 3.5 g/L and 93%, respectively. This represents the highest secretory production of heme-containing proteins ever reported	[80]
Determining the regulation of ligand binding imparted by the proximal and distal heme pockets	Spectroscopic and kinetic characterization of wild-type and mutant LegH	The LegH proximal heme pocket exhibits a stronger bond with bound oxygen compared to myoglobin. While myoglobin relies on additional distal pocket stabilization to lower oxygen dissociation, LegH avoids distal oxygen stabilization to prevent an excessively low dissociation rate constant. These differences in regulatory mechanisms for oxygen binding and dissociation demonstrate how LegH and myoglobin, despite their structural similarities, employ distinct strategies to control their rate constants and oxygen affinities. These strategies are tailored to meet the specific demands of their respective biological environments, allowing them to effectively facilitate oxygen diffusion	[81]

### 4.2 (Plant)ferritin

FT exists in most plants and animals as a molecule consisting of 24 polypeptide subunits – FT heavy chain (FTH) or light type (FT light chain [FTL]). FT, consisting of 36 subunits, is also found in cardiac and skeletal muscle cells. Depending on the origin, the size of a single subunit is approximately 20 kDa (mammals) to 25–28 kDa (plants). In bacteria, FT exists as a molecule consisting of 12 subunits. The structure of the FT molecule is a protein shell filled with an iron core [82]. Plant FT is similar to the animal variety in both function and structure [83].

FT is a protein that stores Fe^3+^ iron ions in the cell. The FTH subunit exhibits iron oxidase activity, oxidizing it from the Fe^2+^ form to Fe^3+^. The FTL subunit, thanks to its nucleation property, leads to the formation of an iron core. In the case of animals, FT in cells of organs involved in iron storage processes (e.g. liver, spleen) is more rich in FTL subunits [84]. FT in cells maintains iron concentration at the level necessary for proper functioning (10^−3^–10^−5 ^M). A single FT molecule has the ability to accumulate up to 10^−2 ^M of iron. Typically, the iron content in a single molecule is less than 3,000 atoms [85]. Most cellular iron is stored in FT. In addition to its iron storage function, FT is also crucial in the processes that protect the cell against excess iron. Excessive amounts of iron at the cellular level may lead to the accumulation of reactive oxygen species (ROS) and oxidative damage; FT prevents such effects by binding free iron [86]. This protein also has a function in cell detoxification by directly participating in the regulation of cellular concentrations of transition metals and other metal ions apart from iron. In this way, FT limits the formation of ROS and alleviates their impact on cellular structures and macromolecules [87]. FT also plays an important role in defense against pathogen attacks. Overexpression of FT as a result of biotic stress causes complexation of iron circulating in the host body – iron becomes unavailable for the life processes of pathogens [88–90]. There is also a correlation between the FT content and the occurrence of autoimmune diseases and inflammatory conditions. Serum FT levels in patients with rheumatoid arthritis may be within the normal range, but synovial fluid and synovial cells have been shown to have elevated FT levels [91]. FT levels decrease during therapy, and FT levels help guide physicians in using glucocorticoids [92]. It has been shown that a high serum FT level is an independent risk factor for severe COVID-19 infection. Assessment of serum FT levels during hospitalization may be helpful in identifying people at high risk of severe COVID-19 disease [93,94].

FT of plant origin is considered important for assessing the possibility of its use as a source of iron in the diet [95–99]. FT from legumes is particularly popular as a source of iron [100]. The bioavailability of a protein of this origin is comparable to FeSO_4_ [87,101]. It is assumed that the absorption of iron from FT takes place by a mechanism independent of the process of iron absorption from FeSO_4_. Studies labeled ^59^Fe derived from FT demonstrated the bioavailability of FT iron as a feature independent of the simultaneous administration of an up to 9-fold higher dose of the mineral form in iron sulfate [102]. The likely mechanism of uptake is AP2 peptide-dependent endocytosis [103].

However, the stability of FT is ambiguously assessed. Selected studies on FT bioavailability and safety are presented in Table 3. Many authors believe that this protein is characterized by very good stability also under digestive conditions (extreme pH values) [87,104,105]. According to other experiments, FT is sensitive to gastric pH (values 1–2), leading to structural changes in the protein. However, these changes may be reversible, and the molecule returns to its native form as the pH in the intestines increases [106]. The food matrix may have a positive effect on maintaining FT stability under digestive conditions [107].

**Table 3:** Review of selected studies on the bioavailability and safety of plant FT

Aim of research	Methods	Conclusions	Refs.
Effects of dietary factors on iron uptake from FT by Caco-2 cells	– Cell culture – *In vitro* digestion of Ft – Treatment of Caco-2 cells with dietary factors and Fe – as FT or ferrous sulfate – Western blots of undigested vs. digested FT	Fe from undigested FT is absorbed by intestinal cells, and this process appears to be minimally affected by common dietary factors. However, once FT is digested, the mineralized core releases Fe, allowing for interactions with dietary factors that can influence absorption. This suggests that while dietary factors can impact the absorption of FT-bound Fe to some extent, their effect is likely comparable to or less than the influence they exert on Fe uptake from more traditional forms of iron supplementation, such as FeSO_4_. It’s worth noting that FeSO_4_, while bioavailable, can lead to undesirable changes in the color and organoleptic properties of fortified products. In contrast, FT-bound Fe, encapsulated within a mineral core and protected by a protein shell, minimizes Fe-induced oxidative damage to food products. This finding highlights the potential of using FT for biofortification of staple foods, as it offers a bioavailable source of Fe that is less prone to compromising product quality. In summary, results suggest that FT is a promising candidate for addressing iron deficiency, offering a bioavailable and food-friendly source of this essential nutrient	[101]
Assessment of soy FT absorption in nonanemic women	Subjects were randomly assigned to start with either a ^59^Fe-labeled soybean FT meal or a ^59^Fe-labeled FeSO_4_ meal, each containing 1 mg Fe, given with 60 mL of apple juice. They consumed a bagel with cream cheese after fasting for 12 hours overnight	The iron status of the 16 women in the study was generally adequate, with only one participant showing slight anemia and four exhibiting ID based on low serum FT concentrations. The mean concentrations of hemoglobin and serum FT fell within expected ranges. Interestingly, there were no significant differences observed in iron absorption between the two different forms of iron or the two methods used for assessment. Both whole-body counting and RBC incorporation methods provided consistent estimates of iron absorption for both soybean FT and FeSO_4_. Furthermore, a notable inverse correlation was observed between serum FT levels and iron absorption, regardless of the source of iron. These findings highlight the complex relationship between serum FT and iron absorption, which appears to be consistent across different iron sources and assessment methods	[104]
	^59^Fe incorporation into red blood cells (RBCs) was measured, and after a blood draw, they received the second randomized labeled meal. Radioactivity was measured in a whole-body counter on days 14 and 28 after the second meal. A final blood sample was taken on day 28. Whole-body iron absorption was calculated based on RBC count in 5 mL of blood, assuming a blood volume of 71.4 mL/kg body weight and an 85% incorporation rate into hemoglobin		
Evaluation of soybean FT intake	*In vitro* by using human intestinal (Caco-2) cells and *in vivo* by using radiolabeled FT and whole-body counting in human subjects	The research highlights that iron from soybeans is readily absorbed, as supported by multiple human studies. This absorption can occur through receptor-mediated endocytosis of intact FT or via DMT1 as ferrous iron, either released from digested FT or potentially from the FT iron core. Increasing the FT content in soybeans holds promise as a sustainable strategy to enhance iron absorption, particularly in vulnerable populations with ID. This avenue of investigation could contribute significantly to addressing iron-related health challenges	[108]
Evaluation of gastric digestion of pea FT and modulation of its iron bioavailability by ascorbic and phytic acids in Caco-2 cells	*In vitro* digestion/Caco-2 cell model in the presence or absence of ascorbic acid and phytic acid	The study demonstrates that pea FT formation significantly increased iron bioavailability compared to the blank digest, with ascorbic acid enhancing and phytic acid decreasing its availability. However, even with ascorbic acid, the FT content in Caco-2 cells was significantly lower with pea FT compared to FeSO_4_. Moreover, under gastric pH conditions, no FT bands were observed in the presence of pepsin, indicating structural instability. Gel filtration chromatography and circular dichroism spectroscopy revealed pH-dependent loss of quaternary and secondary structure in pea FT. These findings suggest that the release of iron from pea FT interacts with dietary factors, ultimately affecting its bioavailability in a manner reminiscent of non-heme iron	[109]
Evaluation of iron absorption from FT	– Radiolabeled materials – Human studies – Animal studies	The study found that a nineold excess of ferrous sulfate did not impact the absorption of iron from FT. This suggests that iron in FT is not absorbed by the same mechanism as ferrous sulfate, and the absorption of FT iron remained unaffected even when saturation kinetics were maintained. Additionally, unlabeled ferrous sulfate had no significant effect on the absorption of ^59^Fe in FT when administered at a ratio of 9:1 (4.5 mg FeSO_4_ and 0.5 mg ^59^Fe as FT iron) in non-gastric-resistant capsules	[102]

Based on the literature data presented, it can be concluded that the use of plant FT allows to provide absorbable iron, but the bioavailability depends strongly on the diet and compounds consumed in the diet that can bind iron with FT. Moreover, it should be mentioned that the use of plant sprouts containing FT in the recipes of food products may cause unfavorable changes in the appearance of these products, which may translate into their lower acceptance among consumers. However, properly thought-out ingredients of vegan products that can be enriched with FT will enable the development of a full-value product with high bioavailability and attractiveness.

## 5 Limitation and future perspectives

This review discusses the properties of LegH and FT as sources of Fe, as well as their role in the body. The use of new sources of iron in plant diets seems, on the one hand, necessary, but on the other hand, it poses many new risks both from the point of view of safety and bioavailability. Many countries do not allow the marketing of food containing ingredients subject to genetic modification or obtained from new GM organisms. For this reason, it seems that FT is a much more promising source of Fe for food fortification for vegans and vegetarians. Nevertheless, it is worth clearly pointing out the need to carefully analyze such enriched food, especially in the context of long-term consumption. Long-term analyses can provide valuable data that may indicate the benefits of using novel sources of Fe.
